# Rapid identification of endometrial hyperplasia and endometrial endometrioid cancer in young women

**DOI:** 10.1007/s12672-023-00736-w

**Published:** 2023-07-03

**Authors:** Dan Kuai, Qingtao Tang, Wenyan Tian, Huiying Zhang

**Affiliations:** 1 Department of Gynecology and Obstetrics, Tianjin Medical University General Hospital, NO 154, Anshan Road, He Ping District, Tianjin, 300052 People’s Republic of China; 2 Tianjin Key Laboratory of Female Reproductive Health and Eugenics, Tianjin, 300052 People’s Republic of China

**Keywords:** Endometrial hyperplasia, Young female, Endometrial cancer, Risk factor, Nomogram

## Abstract

**Purpose:**

We investigated endometrial hyperplasia (EH) and endometrial endometrioid cancer (EEC) and developed a nomogram model to predict the EH/EEC risk and improve patients’ clinical prognosis.

**Methods:**

Data were collected from young females (age: ≤ 40 years) who complained of abnormal uterine bleeding (AUB) or abnormal ultrasound endometrial echoes. The patients were randomly divided into training and validation cohorts at a 7:3 ratio. The risk factors for EH/EEC were determined through the optimal subset regression analysis and a prediction model was developed. We used the concordance-index (C-index), and calibration plots in the training and validation sets to assess the prediction model. We drew the ROC curve in the validation set and calculated the area under the curve (AUC), as well as its accuracy, sensitivity, specificity, negative predictive value, and positive predictive value, and finally, converted the nomogram into a web page dynamic nomogram.

**Results:**

Predictors included in the nomogram model were body mass index (BMI), polycystic ovary syndrome (PCOS), anemia, infertility, menostaxis, AUB type, and endometrial thickness. The C-index of the model in the training and validation sets were 0.863 and 0.858. The nomogram model had good discriminatory power and was well-calibrated. According to the prediction model, the AUC of EH/EC, EH without atypia, and AH/EC were 0.889, 0.867, and 0.956, respectively.

**Conclusions:**

The nomogram of EH/EC is significantly associated with risk factors, namely BMI, PCOS, anemia, infertility, menostaxis, AUB type, and endometrial thickness. The nomogram model can be used to predict the EH/EC risk and rapidly screen risk factors in a women population with high risk.

## Introduction

Endometrial hyperplasia (EH) involves an increase in the number of endometrial linings due to structural changes in endometrial glands and an imbalance (> 1:1) of glands and stroma [1]. EH is associated with the risk of progression to endometrial endometrioid cancer (EEC). The incidence of EH is three times that of EEC [2]. In 2014, the World Health Organization (WHO) categorized EH into EH without atypia and EH with atypia (AH). In the 2003 classification, EH is further classified into simple EH (SH) and complex EH (CH) [3]. A meta-analysis [4] revealed that in SH patients, the probability of EEC development was 1%, and in CH patients, the probability was > 3%. The American College of Obstetricians and Gynecologists Committee [5] reported that in AH patients, approximately 25–40% of patients experience EEC simultaneously, and the EEC risk was increased by 14–45 times.

EH/EEC patients may present with abnormal uterine bleeding (AUB). Among AUB types, irregular uterine bleeding is the most common and may also present symptoms of menostaxis and oligomenorrhea. Long-term estrogen exposure without progesterone antagonism is currently believed to be the leading cause of EH, and polycystic ovary syndrome (PCOS), obesity, diabetes, and infertility are also risk factors. With the increased adaptation of unhealthy lifestyles, and the development of obesity and chronic diseases at a younger age, the EEC incidence is increasing, and EEC also occurs more often in young women. Studies have reported that approximately 25% of EEC patients were premenopausal and 5–10% of the EEC patients were women younger than 40 years [6–8]. EH/EEC severely damages the reproductive and birth health of young women. Consequently, earlier identification, diagnosis, and prevention of EH/EEC are essential for the population of young women.

## Patients and methods

### Patients

The study was approved by the Ethics Committee of Tianjin Medical University General Hospital. We included participants (1) aged ≤ 40 years at the time of consultation; (2) who complained of AUB or abnormal endometrial echoes (including endometrial thickening and cavity occupancy); (3) who had undergone transvaginal ultrasound (TVUS), hysteroscopy, and histopathological examination at Tianjin Medical University General Hospital; and (4) whose pathological diagnoses were EH without atypia, AH/EEC, secretory or proliferative phases of the endometrium. We excluded patients (1) who were being treated with hormone replacement therapy or ovulation induction drugs; (2) who had EH without atypia or AH/EEC along with other malignant tumors; (3) who had a pregnancy and suspected pregnancy; (4) whose clinical and pathological data were missing; and (5) who were in the menopausal stage.

Based on the inclusion and exclusion criteria, 495 patients were finally included from June 1, 2018, to July 31, 2021. Of the 495 patients, 231 patients had EH without atypia, 74 patients had AH and EEC, and 190 patients had a normal endometrium. A normal endometrium was defined as a pathological diagnosis of secretory and proliferative phases of the endometrium). The medical history and physical examination results of EH/EEC patients were carefully recorded. The endometrial thickness was evaluated using TVUS before hysteroscopic localization of endometrial biopsy, followed by histopathological examination.

### Clinical evaluation

#### Demographic and clinical characteristics

Demographic and clinical characteristics including patient age, body mass index (BMI); parity; gravidity; chief complaint; histories of hypertension, type II diabetes, insulin resistance, PCOS, anemia and infertility; menstrual history; and bleeding description were recorded. AUB was categorized as irregular menses (menstrual cycle, menstruation, and amount of menses are abnormal) and other AUB types (including intermenstrual bleeding, menostaxis, hypermenorrhea, and hypomenorrhea).

#### Transvaginal ultrasound

Two sonographers with experience in obstetrics and gynecology and specialized in TVUS interpretation performed TVUS. When the results were inconsistent, a third sonographer was consulted to finalize the TVUS report. The TVUS results described the presence of an endometrial mass (measured by the size of the mass), endometrial stripe thickness (in millimeters), abnormal flow, etc.

#### Pathological diagnoses

Pathological diagnoses were made by gynecological pathologists from the Pathology Department of Tianjin Medical University General Hospital. The most severe pathological diagnosis was considered the final study diagnosis. Based on the results of pathological diagnoses, patients were divided into Group A (normal endometrium), Group B (EH without atypia), and Group C (AH/EEC). A normal endometrium was defined as a pathological diagnosis of secretory and proliferative phases of the endometrium.

### Statistical analysis

The patients were randomly divided into a training cohort (build model) and a validation cohort (validate the model) at a 7:3 ratio. Data were analyzed using the Shapiro–Wilk test and the data conforming to the normal distribution were expressed as the mean ± standard deviation (x ± s). Using one-way ANOVA tests for continuous variables, the Bonferroni method was applied for comparing multiple groups. Data not conforming to the normal distribution were expressed as the median (interquartile range), and the nonparametric test (Kruskal–Wallis rank sum) was used for comparison between groups. Dunnett's method was used for further comparison among multiple groups, and Pearson’s chi-square (χ^2^) or Fisher’s exact test was used for categorical variables.

The optimal subset regression analysis was used to select variables for inclusion in the nomogram which based on the Akaike information criterion minimum [9]. The concordance index (C-index), area under the time-dependent receiver operating characteristic curve (AUC), accuracy, sensitivity, specificity, negative predictive value (NPV), and positive predictive value (PPV) were used to evaluate model [10]. P < 0.05 (two-tailed) was considered statistically significant. All statistical analyses were performed using the R programming language and environment (v.4.1.1; http://www.r-project.org/).

## Results

### Patient characteristics

A total of 495 patients with postoperative pathological findings confirming EH/EEC and a normal endometrium were included. The experimental group (305 cases) was diagnosed with EH without atypia (231 cases) and AH/EEC (74 cases). The control group (190 cases) was diagnosed with a normal endometrium. No difference in demographic and clinical characteristics was observed between the training and validation cohorts (P > 0.05) (Table 1).

**Table 1:** Demographic and clinical characteristics of patients in training and validation cohort

Characteristic	Training cohort (n = 346)	Validation cohort (n = 149)	F/H*/χ*^*2*^	*P value*
Basic situation				
Age (years)	33.35 ± 4.26	33.02 ± 4.33	0.628	0.429
Parity(time)			10.499	0.779
0	154 (44.5%)	70 (47.0%)		
1	86 (24.9%)	38 (25.5%)		
≥ 2	106 (30.6%)	41 (27.5%)		
Gravidity(time)			0.026	0.873
0	204 (59.0%)	89 (59.7%)		
≥ 1	142 (41.0%)	60 (40.3%)		
BMI(kg/m^2^)	25.67 ± 5.49	25.96 ± 5.65	0.293	0.589
Comorbidities				
Hypertension	17 (4.9%)	9 (6.0%)	0.266	0.606
Type II diabetes	13 (3.8%)	9 (6.0%)	1.278	0.258
Insulin resistance	50 (14.5%)	22 (14.8%)	0.008	0.928
PCOS	109 (31.5%)	52 (34.9%)	0.547	0.459
Infertility	79 (22.8%)	38 (25.5%)	0.412	0.521
Anemia	59 (17.1%)	21 (14.1%)	0.673	0.412
Menstrual history				
Menstrual regularity			0.044	0.834
Yes	224 (64.7%)	95 (63.8%)		
No	122 (35.3%)	54 (36.2%)		
Menstrual cycle			0.333	0.564
21–35 days	239 (69.1%)	99 (66.4%)		
> 35 days	107 (30.9%)	50 (33.6%)		
Menstruation			0.017	0.898
3–7 days	291 (84.1%)	126 (84.6%)		
> 7 days	55 (15.9%)	23 (15.4%)		
Menstrual volume			0.055	0.973
5–80 ml	323 (93.4%)	139 (93.3%)		
< 5 ml	6 (1.7%)	3 (2.0%)		
> 80 ml	17 (4.9%)	7 (4.7%)		
Bleeding description			0.538	0.764
Without bleeding	54 (15.6%)	25 (16.8%)		
Other AUB	104 (30.1%)	40 (26.8%)		
Irregular menses	188 (54.3%)	84 (56.4%)		
Duration of AUB (months)	12 (2–60)	12 (2–48)	26.436	0.657
Transvaginal ultrasound				
Endometrial stripe thickness(mm)	12.21 ± 6.38	12.71 ± 8.17	0.534	0.465
Endometrial mass	145 (41.9%)	63 (42.3%)	0.006	0.938
Pathology diagnoses*			2.42	0.304
Normal endometrium	129 (37.3%)	61 (40.9%)		
EH	169 (48.8%)	62 (41.7%)		
AH/EC	48 (13.9%)	26 (17.4%)		

^*^EH endometrial hyperplasia without atypia, *AH* endometrial hyperplasia with atypia, *EC* endometrial cancer

The proportion of nulliparous or null gravidity patients was significantly higher in Group C patients than in Group A patients (P < 0.05). Meanwhile, with an increase in the EH level, the proportion of patients with a higher BMI level, hypertension, diabetes, insulin resistance, PCOS, infertility, and anemia increased gradually (P < 0.05). Compared with patients with normal endometrium, most EH patients had irregular menstruation, oligomenorrhea, and menostaxis (P < 0.05). In total, 95.8% of AH patients complained of irregular menses, which was higher than that in Group A or B. Moreover, their AUB duration was also higher than that in Group A or B (P < 0.05). The endometrial stripe thickness was greater in Group C than in Group A or B. The proportion of TVUS-indicated intrauterine masses was significantly higher in Group C patients than in Group B and A patients (P < 0.05) (Table 2).

**Table 2:** Demographic and clinical characteristics of EH/EC patients

	Group A (n = 129)	Group B (n = 169)	Group C (n = 48)	F/H*/χ2*	*P value*
Basic situation					
Age (years)	33.10 ± 4.19	33.41 ± 4.26	33.83 ± 4.51	0.544	0.581
Parity (time)				30.676	< 0.001
0	42 (32.6%)	80 (47.3%)	32 (66.6%)		
1	29 (22.4%)	43 (25.4%)	14 (29.2%)		
≥ 2	58 (45.0%)	46 (27.3%)	2 (4.2%)		
Gravidity (time)				16.140	< 0.001
0	70 (54.3%)	93 (55.0%)	41 (85.4%)		
≥ 1	59 (45.7%)	76 (45.0%)	7 (14.6%)		
BMI(kg/m^2^)	23.12 ± 3.85	26.31 ± 5.22	30.25 ± 6.53	38.717	< 0.001
Comorbidities					
Hypertension	0 (0.0%)	13 (7.7%)	4 (8.3%)	10.661	0.006
Type II diabetes	1 (0.8%)	9 (5.3%)	3 (6.3%)	5.859	0.041
Insulin resistance	4 (3.1%)	30 (17.8%)	16 (33.3%)	28.775	< 0.001
PCOS	10 (7.8%)	65 (38.5%)	34 (70.8%)	71.925	< 0.001
Infertility	11 (8.5%)	35 (20.7%)	33 (68.8%)	72.855	< 0.001
Anemia	2 (1.6%)	40 (23.7%)	17 (35.4%)	38.592	< 0.001
Menstrual history					
Menstrual regularity				70.037	< 0.001
Yes	116 (89.9%)	95 (56.2%)	13 (27.1%)		
No	13 (10.1%)	74 (43.8%)	35 (72.9%)		
Menstrual cycle				62.816	< 0.001
21–35 days	118 (91.5%)	105 (62.1%)	16 (33.3%)		
> 35 days	11 (8.5%)	64 (37.9%)	32 (66.7%)		
Menstruation				29.644	< 0.001
3–7 days	125 (96.9%)	134 (79.3%)	32 (66.7%)		
> 7 days	4 (3.1%)	35 (20.7%)	16 (33.3%)		
Menstrual volume				17.667	0.426
5–80 ml	125 (96.8%)	159 (94.1%)	39 (81.2%)		
< 5 ml	2 (1.6%)	3 (1.8%)	1 (2.1%)		
> 80 ml	2 (1.6%)	7 (4.1%)	8 (16.7%)		
Bleeding description				90.839	< 0.001
Without bleeding	38 (29.5%)	14 (8.3%)	2 (4.2%)		
Other AUB	59 (45.7%)	45 (26.6%)	0 (0%)		
Irregular menses	32 (24.8%)	110 (65.1%)	46 (95.8%)		
Duration of AUB (months)	5 (0–12)	12 (3–60)	60 (17.25–120)	45.178	< 0.001
Transvaginal ultrasound					
Endometrial stripe thickness (mm)	9.19 ± 3.22	12.58 ± 5.19	18.73 ± 10.35	49.897	< 0.001
Endometrial mass	47 (36.4%)	70 (41.4%)	28 (58.3%)	6.924	0.031

Group A: normal endometrium; Group B: endometrial hyperplasia without atypia; Group C: endometrial hyperplasia with atypia, endometrial cancer

### Development and validation of a nomogram for predicting EH/AH

**Fig. 1:**
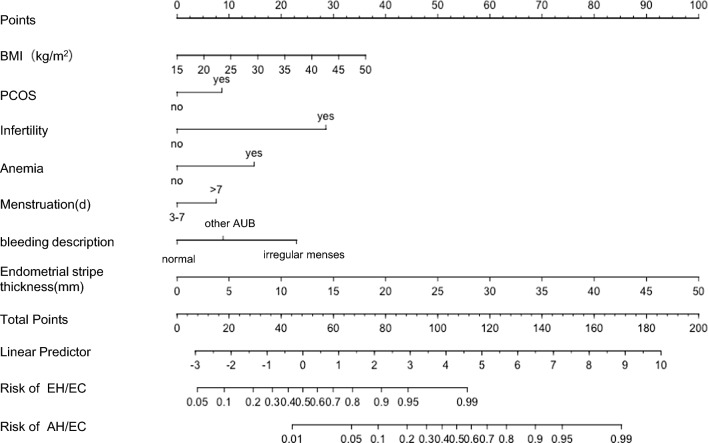
Nomogram prediction model for the risk of developing endometrial hyperplasia and endometrial cancer. The endometrial hyperplasia nomogram prediction models were developed in this retrospective analysis, with BMI level, PCOS, infertility, menstruation, bleeding description, Endometrial stripe thickness. *BMI* body mass index, *PCOS* Polycystic Ovary Syndrome, *AUB* Abnormal Uterine bleeding, *EH* endometrial hyperplasia, *AH* endometrial hyperplasia with atypia, *EC* endometrial cancer

**Fig. 2:**
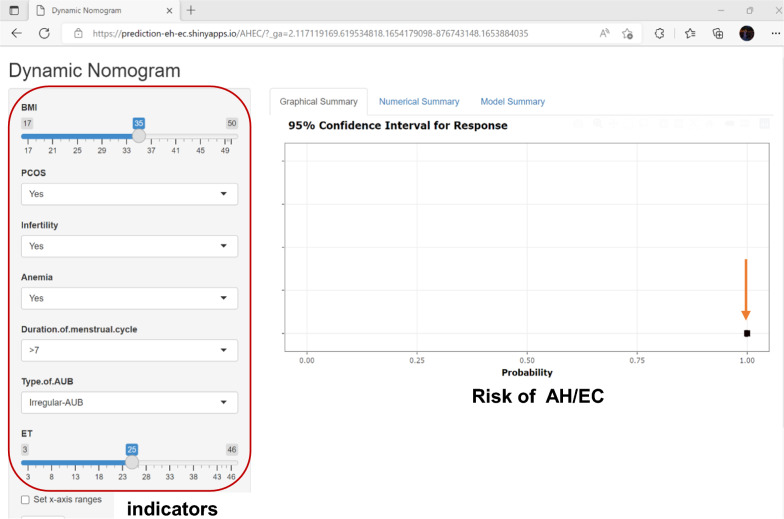
Dynamic Nomogram for AH/EC on web page

The optimal subset regression analysis results revealed that the model containing the BMI level, PCOS, anemia, infertility, menstruation, bleeding description, and endometrial stripe thickness had a minimal AIC value. Patients were scored for predictors, and the scores were summed to obtain a total score that represents the corresponding predicted risk of EH/EEC. The higher the score, the higher the EH/EEC risk. When the total score was ≤ 18 points, the EH/EEC risk was ≤ 10%, and when the total score was ≥ 78 points, the EH/EEC risk was ≥ 90%. When the total score was ≤ 77 points, the AH/EEC risk was ≤ 10%, and when the total score was ≥ 137 points, the AH/EEC risk was ≥ 90% (Fig. 1). Therefore, clinicians should be mindful of identifying the high-risk group for EH/EEC early based on these risk factors. Predict model of high-risk patients can be developed to provide early diagnosis and intervention and to educate patients about symptoms and regular follow-up. To ensure that model application in clinical practice is simpler, we transformed the nomogram into a web-based calculator (https://prediction-eh-ec.shinyapps.io/EHEC/?_ga=2.76675026.619534818.1654179098-876743148.1653884035; https://prediction-eh-ec.shinyapps.io/AHEC/?_ga=2.117119169.619534818.1654179098-876743148.1653884035) (Fig. 2).

### Performance of the nomogram model in the training and validation cohorts

**Fig. 3:**
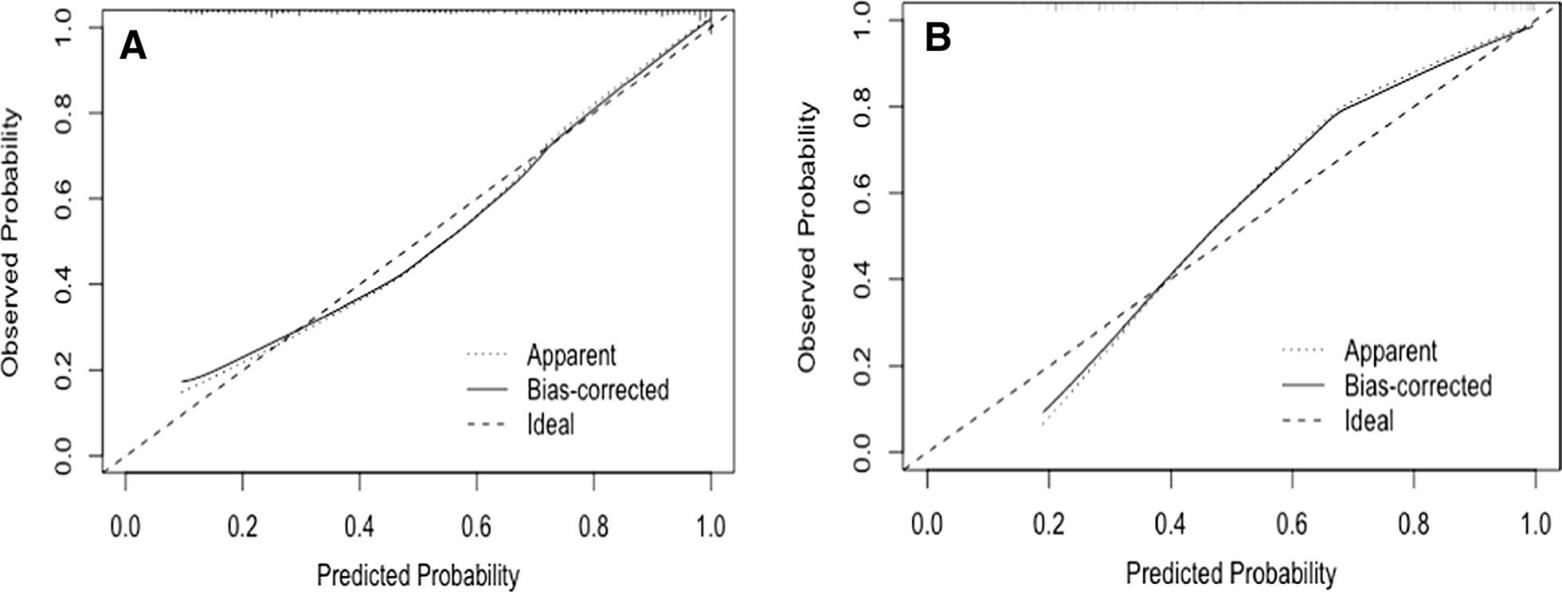
**A**: The calibration curves of nomogram prediction model in training cohort. **B**: The calibration curves of nomogram prediction model in validation cohort. The x-axis indicates the predicted endometrial hyperplasia and endometrial cancer risk. The y-axis indicates the actual diagnosed endometrial hyperplasia and endometrial cancer risk. The diagonal dotted line represents a perfect prediction by an ideal model. The solid line represents the performance of the nomogram, of which a closer fit to the diagonal dotted line represents a better prediction

The model was tested using the Hosmer and Lemeshow goodness of fit test. The χ^2^ value of 1.924, P = 0.382, proved that the model fit goodness was good. The calibration curves exhibited a good agreement across the training set (Fig. 3A). The C-index calculated using the internal verification bootstrap method (1000 times self-sampling) for the model was 0.863.

**Fig. 4:**
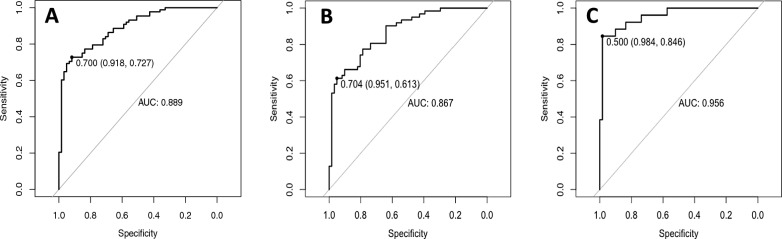
**A**: ROC curves of nomogram predict EH/EC in training cohort. **B**: ROC curves of nomogram predict EH in training cohort. **C**: ROC curves of nomogram predict AH/EC in training cohort. EH/EC: endometrial hyperplasia (endometrial hyperplasia without atypia or endometrial hyperplasia with atypia) and endometrial cancer; AH/EC: endometrial hyperplasia with atypia and endometrial cancer

In the validation cohort, the C-index was 0.858 after 1000 times bootstrap self-sampling internal model verification. The calibration curves displayed a good agreement across the validation set (Fig. 3B). The prediction model predicted that the AUC value of EH/EEC was 0.889, accuracy was 80.53%, sensitivity was 91.80%, specificity was 72.73%, positive predictive value (PPV) was 92.75%, and negative predictive value (NPV) was 70.00%. The AUC value for predicting AH was 0.867, accuracy was 78.05%, sensitivity was 95.08%, specificity was 61.29%, PPV was 92.68%, and NPV was 70.73%. The AUC value for predicting AH/EEC was 0.956, accuracy was 94.25%, sensitivity was 98.36%, specificity was 84.61%, PPV was 95.65%, and NPV was 93.75% (Table 3, Fig. 4).

**Table 3:** Predictive power of Nomogram

	EH/EC	EH	AH/EC
AUC	0.889	0.867	0.956
Accuracy (%)	80.53	78.05	94.25
Sensitivity (%)	91.80	95.08	98.36
Specificity (%)	72.73	61.29	84.61
PPV (%)	92.75	92.68	95.65
NPV (%)	70.00	70.73	93.75

*AUC* the area under the curve, *PPV* positive predictive value, *NPV* negative predictive value, *EH* endometrial hyperplasia without atypia, *AH* endometrial hyperplasia with atypia, *EC* endometrial cancer, *EH/EC* endometrial hyperplasia (endometrial hyperplasia without atypia or endometrial hyperplasia with atypia) and endometrial cancer, *AH/EC* endometrial hyperplasia with atypia and endometrial cancer

## Discussion

EH is a common gynecological, endocrine disease. Risk of progression to EEC exists in the absence of timely intervention. The incidence of EH is 1%–5% in asymptomatic premenopausal women, approximately 10% in premenopausal women with AUB, and approximately 20% in premenopausal women with oligomenorrhea and PCOS [11, 12]. The major clinical manifestations of EH/EEC are irregular vaginal bleeding. Heavy or prolonged bleeding will lead to anemia, thereby affecting other body organs and leading to multiple organ dysfunction. The risk of obesity and chronic metabolic disease increases in young women with unhealthy lifestyles, and EH and EEC continue to occur more often in younger patients, resulting in decreased endometrial receptivity, which further leads to infertility, decreased implantation rate, increased abortion rate, and decreased live birth rate. Moreover, disease-related anxiety and fear can increase physical and mental stress, thereby endangering general health.

The clinical prediction model is a statistical model based on the disease characteristic that is used to predict the probability of a specific outcome event in a population with certain characteristics. At present, an established risk assessment model for EH in young women is lacking, and those available are mostly models for EEC. The model includes the risk factor assessment of EEC by using different factors such as clinical characteristics, magnetic resonance imaging findings, ultrasound findings, and molecular typing results for predicting the presence of myometrial invasion and lymph node metastasis and EEC prognosis. In the study design stage, the relevant published articles were read, and relevant predictive models of EEC were referred to determine the relevant factors and indicators for study inclusion. Nomogram is a form of prediction model that can quickly calculate a patient's disease risk and prognosis and is widely used in clinical practice. Our nomogram is the first prediction model of EH/EEC in young women aged ≤ 40 years and includes seven predictors: BMI level, combined PCOS, combined infertility, combined anemia, menostaxis, AUB type, and endometrial stripe thickness. The model has good calibration and discrimination, and a high C-index for the training and validation sets, which indicates that the model can relatively accurately predict the EH/EEC risk in clinical practice and offer a basis to gynecologists for making clinical decisions.

Obesity is an independent risk factor for EH/EEC. As the BMI level increases, the EH/EEC risk increases. A New Zealand study [13] investigating the association between BMI level and EEC reported that obese women (BMI > 30 kg/m^2^) were four times more likely to develop EH/EEC than women with a normal weight. In a meta-analysis [14] of 26 American Association for Cancer Research studies, with every 5 kg/m^2^ increase in the BMI, the EEC risk increased by 50% [RR: 1.50; 95% confidence interval (CI) 1.42–1.59]. In EEC patients, obesity is often associated with poor prognosis. Compared with women with normal BMI levels, obese women (BMI 30–34.9 kg/m^2^) and morbidly obese women (BMI > 40 kg/m^2^) had RRs of 2.53 and 6.25 for disease-related mortality, respectively [15]. Zhang et al. [10] analyzed the clinical data and serum metabolic results of 205 AH/EEC patients. They included BMI levels, combined hypertension, combined hyperlipidemia, combined hyperglycemia, serum uric acid levels, and CA199 levels as variables to establish a predictive model and drew a nomogram with a C-index of 0.782. Owing to long-term anovulation in PCOS patients, estrogen stimulates the endometrium even in the absence of progesterone antagonism for a long period, leading to EH and even EEC. Because progesterone does not protect the endometrium in patients with infertility, the EH/EEC risk also increases. For patients with high BMI levels, PCOS, and infertility, attention should be paid to the health education of patients. They must be informed of the potential harm of these problems and asked to reduce their weight. Patients with long-term anovulation must use progestins regularly for endometrial protection. In this study, EH/EEC was associated with prolonged menstrual in the menstrual history, with corpus luteum atrophy being a cause of long menstrual duration. This suggests that patients with only long menstrual duration must not be ignored in clinical work. While treating hormone disorders, patients must be informed to regularly monitor endometrial conditions to reduce EH/EEC occurrence.

AUB occurs in 14%–25% of reproductive-age women [16, 17]. AUB is mostly caused by benign lesions, but AUB is also a common clinical symptom of EEC. EH/EEC is the cause of AUB in 5%–14% of AUB patients [18]. Bagepalli Srinivas et al. [19] retrospectively analyzed the clinical data of 472 women with AUB (including 20 with AH and 8 with EEC) who were aged ≤ 55 years and established a predictive scoring model for AH/EC in symptomatic premenopausal women. This model included the variables of age ≥ 45 years (1 point), anovulatory bleeding (3 points), combined diabetes (3 points), and BMI ≥ 30 kg/m^2^ (2 points). The AUC of the model was 0.848 when the score was ≥ 5 points (the optimal cut-off value). The sensitivity, specificity, PPV, and NPV were 85.7%, 87.6%, 30.6%, and 98.9%, respectively. LR + was 6.91 and LR − was 0.16. Jha’s study [20] included 1084 premenopausal (age < 55 years) women with AUB; these women had an endometrial pathology of EH/EEC, which accounted for 4.7%. The variables included in the model included intermenstrual bleeding, BMI > 25 kg/m^2^, endometrial thickness > 13 mm, and hypothyroidism, and the AUC value of the model was 0.971.

Increased endometrial thickness is a risk factor for EH/EEC. TVUS often prompts irregular endometrial thickening or an intrauterine mass in EH/EEC patients. A study [21] of reproductive-age women described the following ultrasound manifestations of EH: endometrial echogenicity, unclear demarcation with the myometrium, increased endometrial thickness, vascularity, uterine cavity effusion, and polypoid masses. The endometrial echogenicity areas and the extent of unclear demarcation with the myometrium were more significant in the AH patients than in the EH patients (P < 0.001). The percentage of combined uterine cavity effusion and the degree of vascular enrichment were also significantly higher in the AH patients than in the EH patients (P < 0.001). The sensitivity, specificity, positive and negative predictive values, and ultrasound accuracy for predicting EH were 96%, 85%, 82%, 94%, and 84%, respectively. Cong et al. [22] analyzed patients with an abnormal endometrial echo, as indicated on ultrasound. Among 692 patients, 55.20% (382/692) of the patients had a normal endometrium, and 44.80% (310/692) of the patients had endometrial lesions; of them, 39.31% (272/692) were benign lesions. Of the 692 patients, 5.49% (38/692) had AH/EEC. Multivariate regression analysis revealed that age ≥ 50 years, endometrial thickness ≥ 7 mm, and postmenopausal bleeding were risk factors for AH/EEC, and the AUC value of the model was 0.771. In total, 57 patients with amenorrhea and anovulation were included in Tingthanatikul’ s study. EH accounted for 45.6%, and 1 case was AH. An endometrial hyperechogenic pattern was the only clinical predictor of EH with borderline significance [23]. Kim et al. [24] enrolled 162 premenopausal and perimenopausal women with AUB or an abnormal endometrium, as indicated on ultrasound. In 14 of the 162 patients who were pathologically EH + (10 EH, 3 AH, and 1 EEC), endometrial stripe abnormality was significantly associated with EH + (P = 0.003) and marginally associated with AEH + (P = 0.05).

In addition, there are a number of models related to EH. Giannella et al. [18] investigated 240 premenopausal women with AUB (including 12 EH/EEC cases) and established a predictive model for EH/EEC. The variables included were BMI ≥ 30 kg/m^2^, diabetes, and endometrial thickness > 11 mm, and the AUC value of the model was 0.854. By enrolling 1369 AUB patients, including 167 AH/EEC patients, Ruan et al. [25] constructed a prediction model of AH/EEC. The final included indicators were metabolic diseases, family history, age ≥ 40 years, ultrasonic blood flow RI ≤ 0.5, and ET ≥ 10 mm, and the AUC value of the model was 0.837. The study population of Bagepalli [19], Jha [20], Giannella [18], Ruan [25], and Tingthanatikul [23] was AUB patients. Our study targeted young women (age < 40 years), patients with endometrial abnormalities, as suggested by AUB or TVUS, were studied, and therefore, the results have a wider range of clinical applicability. At the same time, our study included a larger number of EH/EEC patients and more comprehensive related indicators. AUB patterns, such as irregular uterine bleeding, and ultrasound findings, such as abnormal endometrial thickening, suggest a high EH/EEC risk. Detailed medical history of such patients must be recorded at the first visit, and the general condition of the patients should not be ignored. The nomogram is quickly used to evaluate young women and predict the probability of EH/EEC. Some patients undergo endometrial biopsy owing to the lack of sexual life or fear of hysteroscopy/curettage. In these patients, the predicted probability can be used to communicate with them, so as to reduce any delay in EH/EEC diagnosis and treatment, protect the reproductive health of young women, and improve their reproductive outcomes.

In our study, chronic diseases such as diabetes, hypertension, and insulin resistance were not included among the final predictors. The prevalence of chronic diseases, such as hypertension and diabetes, is positively correlated with age. As individuals age, the prevalence rate of chronic diseases in the population becomes higher. In this study, the participants were young women aged ≤ 40 years, and the overall incidence of chronic diseases, such as hypertension and diabetes, was low among these young patients. Among the 495 study patients, 26 (5.2%) were female patients with hypertension (1 patient with normal endometrium, 19 EH patients, and 6 AH/EEC patients). Twenty-two female patients had diabetes mellitus (4.4%), including 3 patients with normal endometrium, 14 EH patients, and 5 AH/EEC patients. Because of the low proportion of combined hypertension and diabetes, no significant difference was observed between the groups in the statistical analysis owing to the small sample size; therefore, hypertension and diabetes were not included in the final model. However, among patients with hypertension and diabetes, the proportion of patients with a pathological type of EH/EEC was higher than that of patients with a normal endometrium. Hypertension and diabetes may also act as risk factors for EH/EEC in young women. At the same time, the patients with hypertension and diabetes were older in this study. The sample size can be expanded in the later stage to reduce such errors. Therefore, women with risks of EH and EEC should be reminded to improve their lifestyles and be aware of the occurrence and development of chronic diseases.

From 1970 to 2003, JV Lacey et al. [26] enrolled 138 patients who developed cancer at least 1 year (median 6.5 years) after EH diagnosis. Compared with abnormal endometrial proliferation, AH was associated with a significantly increased cancer risk (RR: 14, 95% CI 5–38). The risk was highest at 1–5 years after AH diagnosis (RR: 48, 95% CI 8–294) and remained elevated at 5 years (RR: 3.5, 95% CI 1.0–9.6). This study mainly focused on the risk factor assessment for EH and EEC and the prediction of the EH and EEC risks. The study participants were young women aged < 40 years, with most of them having fertility needs. At the same time, some patients may refuse endometrial biopsy because of lack of sexual life; therefore, predicting and assessing the EH/EEC risk in these patients is necessary as much as possible to reduce missed diagnoses, prevent delay in treatment, and protect the reproductive health of young women. Moreover, our team will follow by patients with EH/EEC, and the main observations were the relief, recurrence, progress, or partial relief of the disease, and the outcome of the patient's pregnancy, and the inclusion of additional patients and factors that may be related to prognosis, and the factors associated with EH/EEC.

According to the recommendation of the transparent reporting of a multivariable prediction model for individual prognosis or diagnosis statements [27]. The favorable results were replicated well in the validation cohort. Our nomogram may be valuable for evaluating EH/EEC patients. When the total score was ≤ 18 points, the EH/EEC risk was 10%, and when the score was ≥ 78 points, the EH/EEC risk was ≥ 90%. When the score was ≤ 77 points, the AH/EEC risk was 10%, and when the score was ≥ 137 points, the AH/EEC risk was ≥ 90%. The C-index values were 0.863 and 0.858 for the training and validation cohorts. The AUC values for predicting EH/EEC, EH, and AH/EEC were 0.889, 0.867, and 0.956, respectively, which indicated that the accuracy and efficiency of the model were good and could provide a basis for clinical decision-making.

### Limitations

This is a retrospective single-center study. To reduce the selection bias, three gynecology experts and one statistics expert were invited to strictly define the inclusion and exclusion criteria. All our patients were younger than 40 years, were admitted to our hospital from June 2016 to June 2021, and had pathological results of EH, EEC, and normal endometrium. The completeness of medical history and auxiliary examination data was determined one by one. For partial data or data missing in the medical records, the patients were followed up to complete the medical data as much as possible. As many participants as possible were included in the study to reduce bias. During the statistical analysis, the included participants were randomly divided into training and validation cohorts. The optimal subset regression was used to reduce the bias-induced influence of confounding factors as much as possible. However, the selection bias cannot be completely controlled by these measures, because hospitals have different criteria for patient admission and patients have different criteria when getting admitted to a hospital. Different diseases and admission conditions make the case and control groups lack comparability, and therefore, the study cases or controls are not representative of the relevant population. In the future, multicenter hospitals (local hospitals, hospitals at all levels) should be included with more participants to reduce the selection bias as much as possible.

## Conclusions

Given its increased accuracy, good clinical utility, and more precise prognosis prediction, our nomogram may be used to predict the risk of EH/EEC patients.

## Data Availability

The data that support the findings of this study are available from the corresponding author upon reasonable request.

## Code availability

The datasets generated during and/or analyzed during the current study are available from the corresponding author upon reasonable request.

## References

[1] Sanderson PA, Critchley HO, Williams AR, Arends MJ, Saunders PT (2017). New concepts for an old problem: the diagnosis of endometrial hyperplasia. Hum Reprod Update.

[2] Reed SD, Newton KM, Clinton WL, Epplein M, Garcia R, Allison K (2009). Incidence of endometrial hyperplasia. Am J Obstet Gynecol.

[3] Sobczuk K, Sobczuk A (2017). New classification system of endometrial hyperplasia WHO 2014 and its clinical implications. Prz Menopauzalny.

[4] Doherty MT, Sanni OB, Coleman HG, Cardwell CR, Mccluggage WG, Quinn D (2020). Concurrent and future risk of endometrial cancer in women with endometrial hyperplasia: a systematic review and meta-analysis. PLoS ONE.

[5] The American College of Obstetricians and Gynecologists Committee Opinion no. 631. Endometrial intraepithelial neoplasia. Obstet Gynecol. 2015;125(5):1272–8. 10.1097/01.AOG.0000465189.50026.20. 10.1097/01.AOG.0000465189.50026.20 25932867

[6] Carneiro MM, Lamaita RM, Ferreira MC, Silva-Filho AL (2016). Fertility-preservation in endometrial cancer: is it safe? Review of the literature. JBRA Assist Reprod.

[7] Duska LR, Garrett A, Rueda BR, Haas J, Chang Y, Fuller AF (2001). Endometrial cancer in women 40 years old or younger. Gynecol Oncol.

[8] Yamagami W, Aoki D (2015). Annual report of the committee on gynecologic oncology, the Japan society of obstetrics and gynecology. J Obstet Gynaecol Res.

[9] Balachandran VP, Gonen M, Smith JJ, Dematteo RP (2015). Nomograms in oncology: more than meets the eye. Lancet Oncol.

[10] Zhang H, Kong W, Han C, Liu T, Li J, Song D (2021). Correlation of metabolic factors with endometrial atypical hyperplasia and endometrial cancer: development and assessment of a new predictive nomogram. Cancer Manag Res.

[11] Göl K, Saraçoğlu F, Ekici A, Sahin I (2001). Endometrial patterns and endocrinologic characteristics of asymptomatic menopausal women. Gynecol Endocrinol.

[12] Park JC, Lim SY, Jang TK, Bae JG, Kim JI, Rhee JH (2011). Endometrial histology and predictable clinical factors for endometrial disease in women with polycystic ovary syndrome. Clin Exp Reprod Med.

[13] Wise MR, Gill P, Lensen S, Thompson JM, Farquhar CM (2016). Body mass index trumps age in decision for endometrial biopsy: cohort study of symptomatic premenopausal women. Am J Obstet Gynecol.

[14] Report, World Cancer Research Fund/American Institute for Cancer Research: Continuous Update Project. Food: nutrition, physical activity, and the prevention of endometrial cancer. 2018.

[15] Calle EE, Rodriguez C, Walker-Thurmond K, Thun MJ (2003). Overweight, obesity, and mortality from cancer in a prospectively studied cohort of U.S. adults. N Engl J Med.

[16] Fraser IS, Langham S, Uhl-Hochgraeber K (2009). Health-related quality of life and economic burden of abnormal uterine bleeding. Expert Rev Obstet Gynecol.

[17] Munro MG, Critchley HO, Fraser IS (2011). The FIGO classification of causes of abnormal uterine bleeding Malcolm G. Munro, Hilary O.D. Crithcley, Ian S. Fraser, for the FIGO working group on menstrual disorders. Int J Gynaecol Obstet.

[18] Giannella L, Cerami LB, Setti T, Bergamini E, Boselli F (2019). Prediction of endometrial hyperplasia and cancer among premenopausal women with abnormal uterine bleeding. Biomed Res Int.

[19] Bagepalli Srinivas S, Kubakaddi SS, Polisetti S, Amber S, Guruvare S, Vaman Pai M (2020). A novel risk-scoring model for prediction of premalignant and malignant lesions of uterine endometrium among symptomatic premenopausal women. Int J Womens Health.

[20] Jha S, Singh A, Sinha HH, Bhadani P, Anant M, Agarwal M (2021). Rate of premalignant and malignant endometrial lesion in "low-risk" premenopausal women with abnormal uterine bleeding undergoing endometrial biopsy. Obstet Gynecol Sci.

[21] Goncharenko VM, Beniuk VA, Kalenska OV, Demchenko OM, Spivak MY, Bubnov RV (2013). Predictive diagnosis of endometrial hyperplasia and personalized therapeutic strategy in women of fertile age. Epma j.

[22] Cong Q, Luo L, Zhongpeng Fu, Jiaqi Lu, Jiang W, Sui L (2021). Histopathology of women with non-uniform endometrial echogenicity and risk factors for atypical endometrial hyperplasia and carcinoma. Am J Transl Res.

[23] Tingthanatikul Y, Choktanasiri W, Rochanawutanon M, Weerakeit S (2009). Prevalence and clinical predictors of endometrial hyperplasiain anovulatory women presenting with amenorrhea. Gynecol Endocrinol.

[24] Kim MJ, Kim JJ, Kim SM (2016). Endometrial evaluation with transvaginal ultrasonography for the screening of endometrial hyperplasia or cancer in premenopausal and perimenopausal women. Obstet Gynecol Sci.

[25] Ruan H, Chen S, Li J, Ma L, Luo J, Huang Y (2023). Development and validation of a nomogram prediction model for endometrial malignancy in patients with abnormal uterine bleeding. Yonsei Med J.

[26] Lacey JV, Ioffe OB, Ronnett BM, Rush BB, Richesson DA, Chatterjee N (2008). Endometrial carcinoma risk among women diagnosed with endometrial hyperplasia: the 34-year experience in a large health plan. Br J Cancer.

[27] Collins GS, Reitsma JB, Altman DG, Moons KG (2015). Transparent reporting of a multivariable prediction model for individual prognosis or diagnosis (TRIPOD): the TRIPOD statement. BMJ.

